# Mucosal Adjuvant Activity of IL-2 Presenting Spores of *Bacillus subtilis* in a Murine Model of *Helicobacter pylori* Vaccination

**DOI:** 10.1371/journal.pone.0095187

**Published:** 2014-04-17

**Authors:** Krzysztof Hinc, Małgorzata Stasiłojć, Iwona Piątek, Grażyna Peszyńska-Sularz, Rachele Isticato, Ezio Ricca, Michał Obuchowski, Adam Iwanicki

**Affiliations:** 1 Department of Medical Biotechnology, Intercollegiate Faculty of Biotechnology UG-MUG, Medical University of Gdańsk, Gdańsk, Poland; 2 Department of Medical Biotechnology, Intercollegiate Faculty of Biotechnology UG-MUG, University of Gdańsk, Gdańsk, Poland; 3 Tri-City Animal Laboratory, Medical University of Gdańsk, Gdańsk, Poland; 4 Department of Biology, Federico II University of Naples, Naples, Italy; Institut Pasteur Paris, France

## Abstract

The endospores of *Bacillus subtilis* are now widely used as a platform for presentation of heterologous proteins and due to their safety record and high resistance to harsh environmental conditions can be considered as potential vehicles for oral vaccination. In this research we show that recombinant *B. subtilis* spores presenting a fragment of the *Helicobacter acinonychis* UreB protein and expressing the *ureB* gene under vegetative promoter elicit a strong cellular immune response in orally immunized mice when co-administered with spores presenting IL-2. We show for the first time the successful application of two types of recombinant spores, one carrying an antigen and the other an adjuvant, in a single oral immunization.

## Introduction

The display of active molecules on the surface of microorganisms is a promising technology to be used in the biotechnology and medicine [1], [2]. A special attention is paid to bacterial endospores as carriers of heterologous proteins [3], which are advantageous to whole-cell display systems because of their unique properties.

Endospores are dormant forms of bacteria belonging to different genera, but most extensively studied surface display systems are based on *Bacillus subtilis* endospores [4]. *B. subtilis* spores are highly resistant to non-physiological and harsh environmental conditions. Such properties mainly result from the presence of protective structure surrounding spore called the coat. Multilayered coat is formed by at least seventy different proteins (Cot proteins) and composes of an inner and outer coat [5] as well as the outermost layer called the crust [6], [7]. Three coat proteins, CotB, CotC and CotG have been used for display of heterologous enzymes and antigens on the spore surface [8]–[11].

So far *B. subtilis* spores have been successfully used to develop protection in animal models against various pathogens such as *Clostridium perfingens* [12], *Clostridium difficile* [13], *Clostridium tetani* [14] or Rotavirus [15]. In all these examples spore-based vaccines have been delivered by a mucosal route and have been shown to stimulate both systemic and localized immune responses. *B. subtilis* spores have also been shown to induce balanced Th1/Th2 response [16] and could be used as a mucosal adjuvant in some applications [17]. Moreover, taking into account probiotic properties of *B. subtilis* and its spores [18], these features make them very attractive candidates as vaccine carriers, especially in oral immunizations.

*Helicobacter pylori* is a major factor causing chronic gastritis and significantly increases the risk of developing peptic ulcer disease and gastric cancer [19]. Current treatments of *H. pylori* infections are encountering problems caused by antibiotic resistance (especially to metronidazole and clarithromycin) leading to growing difficulties in eradication of this bacterium [20]. Infection with *H. pylori* is related to Th1-biased T-cell response and generally elicits robust cellular and humoral immune responses. In spite of these facts, spontaneous eradication of these bacteria form human body is very rare. Moreover, the research conducted on animal models suggests, that establishing humoral immunity does not protect against infection [19].

Several approaches to the construction of a vaccine against *H. pylori* infections have been undertaken. One of the strategies used subunit A of urease (UreA) as an antigen the use of which has been patented (OraVax Inc., Cambridge, MA, US) and the vaccine based on this protein has been used in clinical studies (phase I) [21]–[23]. Another successful approach to immunization against *H. pylori* infection has been based on multi-epitope DNA vaccine with CpG oligonucleotides and LTB as adjuvants [24]. The results of other trials to immunize mice with *H. pylori oipA* gene-encoded construct co-delivered by IL-2 gene-encoded construct and LTB [25], as well as Salmonella vector construct that expressed fusion proteins complexed with *H. pylori* CagA, VacA and UreB in different arrangements suggested an important role of use of multiple antigen in formulation along with an adjuvant leading to Th1 shift of cellular response [26].

Here we report that recombinant *Bacillus subtilis* spores presenting UreB protein elicit cellular immune response in orally immunized mice when administered along with spores presenting human IL-2. Such formulation seems to be a promising vaccine candidate against *Helicobacter pylori* infections.

## Materials and Methods

### Ethics statement

This study was carried out in strict accordance with the recommendations in the institutional and national guidelines for animal care and use. The protocol was approved by the Committee on the Ethics of Animal Experiments of the Medical University of Gdańsk (Permit Number: 4/2010). All surgery was performed under isoflurane anesthesia, and all efforts were made to minimize suffering.

### Bacterial strains and transformation

*Bacillus subtilis* strains used in this study are listed in Table 1. Plasmid amplifications for nucleotide sequencing and subcloning experiments were performed with *Escherichia coli* strain DH5α [27]. Bacterial strains were transformed by previously described procedures: CaCl_2_-mediated transformation of *E. coli* competent cells [27] and transformation of *B. subtilis* [28].

**Table 1:** Strain list.

Strain	Relevant genotype	Reference
***Escherichia coli***		
DH5á	*fhuA2 lac(del)U169 phoA glnV44 Ö80′ lacZ(del)M15 gyrA96 recA1 relA1 endA1 thi-1 hsdR17*	[27]
***Bacillus subtilis***		
168	*trpC*2	[49]
BKH48	*amyE:: cotC-ureB3*	This work
BKH108	*thrC:: rrnOP2-ureB, amyE:: cotC-ureB3*	This work
BKH121	*thrC::cotB*-linker*-IL-2*	This work

### Construction of gene fusions

DNA coding for CotC coat protein was PCR amplified using the *B. subtilis* chromosome as a template and oligonucleotides pair cotC-F/cotC-R (Table 2) as primers. Amplification product of 383 bp was cloned into the pDL vector [29] obtained from Bacillus Genetic Stock Center yielding plasmid pKH29.

**Table 2:** Oligonucleotide list.

Name	Sequence (5′-3′)	Restriction site
cotB-F	GC**GGATCC**GGATGATTGAT	*Bam*HI
cotB-R	GAT**GAATTC**ACGGATTAGGCC	*Eco*RI
cotC-F	GG**GGATCC**GTAGTGTTTTTTATGC	*Bam*HI
cotC-R	CA**GAATTC**TGTAGGATAAATCGTTTGG	*Eco*RI
ureB-F	GCTAC**GGATCC**AAATACACCATTAACCCAG	*Bam*HI
ureB-R	GCACCT**GAGCTC**TAACTTTTGTTGCTTGAG	*Sac*I
IL-2linker-F	GCTTC**ACATGT**TTACGTCAGTGTAGAGATGATAGATTGGC	*Pci*I
IL-2linker-R	CATAT**GGATCC**GGTGGAGGAGAAGCAGCAGCG	*Bam*HI
rOP2-F	GATGGCT**AAGCTTC**ATGGGTCTCACCTCCTTGTTC	*Hind*III
rOP2-R	GGCGTAGAG**GAATTC**GATCTGCATGACCATTATGAC	*Eco*RI
ureBw-F	CCAT**AAGCTT**AAAAAGATTAGCAGAAAAG	*Hind*III
ureBw-R	CTCC**ACATGT**ATCCTAGAAAATGCTAAAG	*Pci*I
hisureB-F	CTC**GAATTC**GTTGCTCCTAAAAAATCCT	*Eco*RI
hisureB-R	CTATT**GCTAGC**ATGCATCACCATCACCATCACTCGAAAAAGATTAGCAGAAAAGA	*Nhe*I

The recognition sites for the restriction enzymes are in bold.

A 655 bp DNA fragment coding for a fragment of UreB was PCR amplified using *Helicobacter acinonychis* chromosome as a template and oligonucleotides ureB-F and ureB-R (Table 2) as primers. The PCR product was sequentially digested with *BamHI* and *SacI* and cloned in frame to the 3′ end of the *cotC* gene carried by plasmid pKH29 yielding plasmid pKH108.

A plasmid enabling integration of *p_rrnO2_* fusion with *ureB* gene into *thrC* locus was constructed as follows. A 303 bp fragment of *B. subtilis* chromosome containing promoter of *rnnOP* operon was PCR amplified using oligonucleotides rop2-F and rop2-R (Table 2) as primers. The PCR product was sequentially digested with *Hind*III and *EcoR*I and cloned into the pDG1663 vector [30] obtained from Bacillus Genetic Stock Center yielding pKH100 plasmid. Next, a 1730 bp fragment encoding entire UreB protein was PCR amplified using *H. acinonychis* chromosome as a template and oligonucleotides ureBw-F and ureBw-R (Table 2) as primers. Obtained PCR product was sequentially digested with *Hind*III and *Pci*I and cloned into the pKH100 plasmid yielding pKH101 plasmid.

DNA coding for CotB coat protein was PCR amplified using the *B. subtilis* chromosome as a template and oligonucleotides pair cotB-F/cotB-R (Table 2) as primers. Amplification product of 1094 bp was cloned into the pDG1663 vector obtained from Bacillus Genetic Stock Center yielding plasmid pKH117.

A gene encoding human IL-2 with *B. subtilis* optimized codon usage flanked by restriction enzyme sites with the sequence coding for GGGEAAAKGGG peptide linker at the N-terminus was synthesized at Eurogentec (Belgium) and delivered in pUC19 vector. A 457 bp fragment coding for IL-2 with peptide linker at N-terminus was PCR amplified using oligonucleotides IL-2linker-F and IL-2linker-R (Table 2) as primers and the plasmid containing synthetic gene as template. Obtained PCR product was sequentially digested with *BamH*I and *Pci*I and cloned into the pKH117 vector yielding pKH122 plasmid.

### Chromosomal integration

Appropriate plasmids were linearized by digestion with a single cutting restriction enzyme. Linearized DNA was used to transform competent cells of the *B. subtilis* strain 168. In case of pKH108 plasmid chloramphenicol-resistant (Cm^R^) clones were the result of a double-crossover recombination event, resulting in the interruption of the non-essential *amyE* gene on the *B. subtilis* chromosome. Several Cm^R^ clones were tested by PCR using chromosomal DNA as a template and oligonucleotides AmyS and AmyA [31] to prime the reaction. Selected clones were called BKH48 and used for subsequent transformation with linearized pKH122 plasmid. Obtained erythromycin-resistant (Erm^R^) clones were the result of double-crossover recombination in the non-essential *thrC* gene. Several Cm^R^ Erm^R^ clones were tested by PCR. Selected clones were called BKH108 and stored for further experiments.

In case of transformation with linearized pKH122 plasmid the verification of obtained clones followed the same procedure as for the construction of BKH108 strain with selection for erythromycin-resistant colonies. Selected clones were called BKH121 and stored for further research.

### Preparation of spores

Sporulation was induced by the exhaustion method in DS (Difco-Sporulation) medium as described elsewhere [32]. PMSF (0.05 M) was included to inhibit proteolysis. After the final suspension in water spores were treated at 65°C for 1 h to kill any residual cells. The spore suspension was titrated immediately for CFU/ml before freezing at −20°C. By this method we could reliably produce 6×10^10^ spores per litre of DSM culture.

### Spore germination

Spore germination measurements in the presence of l-alanine or AGFK solution were performed as follows. Spores were heat activated at 80°C for 10 min and diluted to an OD_600_ of 1 in 10 mM l-alanine and 10 mM Tris-HCl, pH 7.5 (for l-alanine-induced spore germination) or in 10 mM Tris-HCl at pH 7.5 with 3.3 mM l-asparagine, 5.6 mM d-glucose, 5.6 mM d-fructose, and 10 mM KCl (for AGFK-induced spore germination). Germination was then monitored by following the loss of absorbance of spore suspensions at 600 nm.

### Extraction of spore coat proteins

Spore coat proteins were extracted from 50 µl of a suspensions of spores at high density (1×10^10^ spores per ml) using a decoating extraction buffer as described elsewhere [33]. Extracted proteins were assessed for integrity by SDS-polyacrylamide gel electrophoresis (PAGE) and for concentration by two independent methods: the Pierce BCA Protein Assay (Pierce, USA) and the BioRad DC Protein Assay kit (Bio-Rad, USA).

### Western and dot blotting analyses

Western blotting analyses were performed as described elsewhere [10]. Dot blotting analyses were performed as previously described [11] and followed by densitometric analysis with Chemidoc XRS (Bio-Rad, USA) and the MultiAnalyst software.

### Immunofluorescence microscopy

Samples were prepared as previously described [11]. The coverslip was mounted onto a microscope slide and viewed using a Zeiss Axioplan fluorescence microscope with the same exposure time for all samples. Images were captured using a camera connected to the microscope, processed with Corel Photo-Paint software and saved in TIFF format.

### Purification of UreB and antibody production

The ureB gene of *H. acinonichis* was PCR amplified using chromosomal DNA as a template and oligonucleotides hisureB-up and hisureB-dn (Table 2) as primes. DNA encoding six histidines (His6-tag) was carried by oligonucleotide hisureA-dn. The obtained PCR product of 1730 bp was digested with enzymes *EcoR*I and *Nhe*I and cloned into the commercial vector pBAD (Stratagene). The resulting plasmid, pJK01, was verified by restriction analysis and nucleotide sequencing. The protein was purified and used for antibody production following method described previously [10]

### Immunizations

Five groups of eight mice (female, BALB/c, 8 weeks) were immunized by oral route with suspensions of either spores expressing CotC-UreB3 (BKH108), CotB-linker-IL-2 (BKH121), both CotC-UreB3 and CotB-linker-IL-2 (1∶1) or control, non-expressing, spores (strain 168). A naive, non-immunized control group was included. Oral immunizations contained 1.0×10^10^ spores in a volume of 0.2 ml and were administered by intra-gastric lavage on days 1, 3, 5, 22, 24, 26, 43, 45, 47. Serum samples and spleen were collected on days 1, 22, 43 and 61 from two animals per group.

### Indirect ELISA for detection of antigen-specific serum

Plates were coated with 100 µl per well of the specific antigen (2 µg/ml in carbonate/bicarbonate buffer) and left at room temperature overnight. Antigen was UreB purified protein. After blocking with 0.5% BSA in PBS for 1 h at 37°C serum samples were applied using a two-fold dilution series starting with a 1/20 dilution in ELISA diluent buffer (0.1 M Tris-HCl, pH 7.4; 3% (w/v) NaCl; 0.5% (w/v) BSA; 10% (v/v) sheep serum (Sigma); 0.1% (v/v) Triton-X-100; 0.05% (v/v) Tween-20). Every plate carried replicate wells of a negative control (a 1/20 diluted pre-immune serum), a positive control (serum from mice immunized intraperitoneally with UreB purified protein). Plates were incubated for 2 h at 37°C before addition of anti-mouse AP conjugates (Sigma). Plates were incubated for a further 1 h at 37°C then developed using the substrate pNPP (*para*-Nitrophenylphosphate; Sigma).

Reactions were stopped using 2 M H_2_SO_4_.

### Isolation of splenocytes

Mice were sacrificed and spleen was aseptically removed. The spleens were then perfused with RPMI-1640 (supplemented with 10% heat inactivated fetal calf serum, 25 mM HEPES, 2 mM L-glutamine, 1 mM sodium pyruvate, 100 IU/ml penicillin and 100 µg/ml streptomycin) using 5 ml syringe fitted with 26 G needle to obtain single cell suspension of splenocytes. The splenocytes suspension was then centrifuged at 300×g for 15 min. The RBCs were lysed by hypotonic shock using 3 ml of 0.84% of sterile NH_4_Cl or ACK lysis buffer for 5 min. The cells were then washed thrice with RPMI 1640 to remove lysed RBCs and NH_4_Cl.

### IFN-γ and IL-4 ELISpot assay

The numbers of IFN-γ and IL-4 -secreting cells were determined by using mouse IFN-γ or IL-4 ELISpot respectively kit according to manufacturer's instructions (BD ELISpot). Splenocytes (2×10^5^/mL) were cultured in presence or absence of UreB antigen for 48 h. The spots were counted using automated ELISpot plate reader (CTL-ImmunoSpot S6 Micro Analyzer, USA). ELISpot tests have been performed for each animal in three technical repeats. Results were statistically evaluated using Student's t-test.

## Results

### Construction and chromosomal integration of gene fusions

To obtain recombinant *B. subtilis* spores expressing UreB the coding part of the *ureB* gene of *H. acinonychis* was fused in frame with the coding part of *cotC*. The gene fusion retained the promoter of the *cot* gene to ensure proper timing of expression during the sporulation process. Gene fusions were integrated into the *B. subtilis* chromosome at the non-essential locus *amyE*. As heterologous part we used a fragment of UreB that encompassed 166 amino acids (residues 418 to 584). This fragment of *ureB* gene coding for putative most immunogenic regions was designated with Antigen program (a part of EMBOSS package; http://emboss.sourceforge.net/).

**Figure 1:**
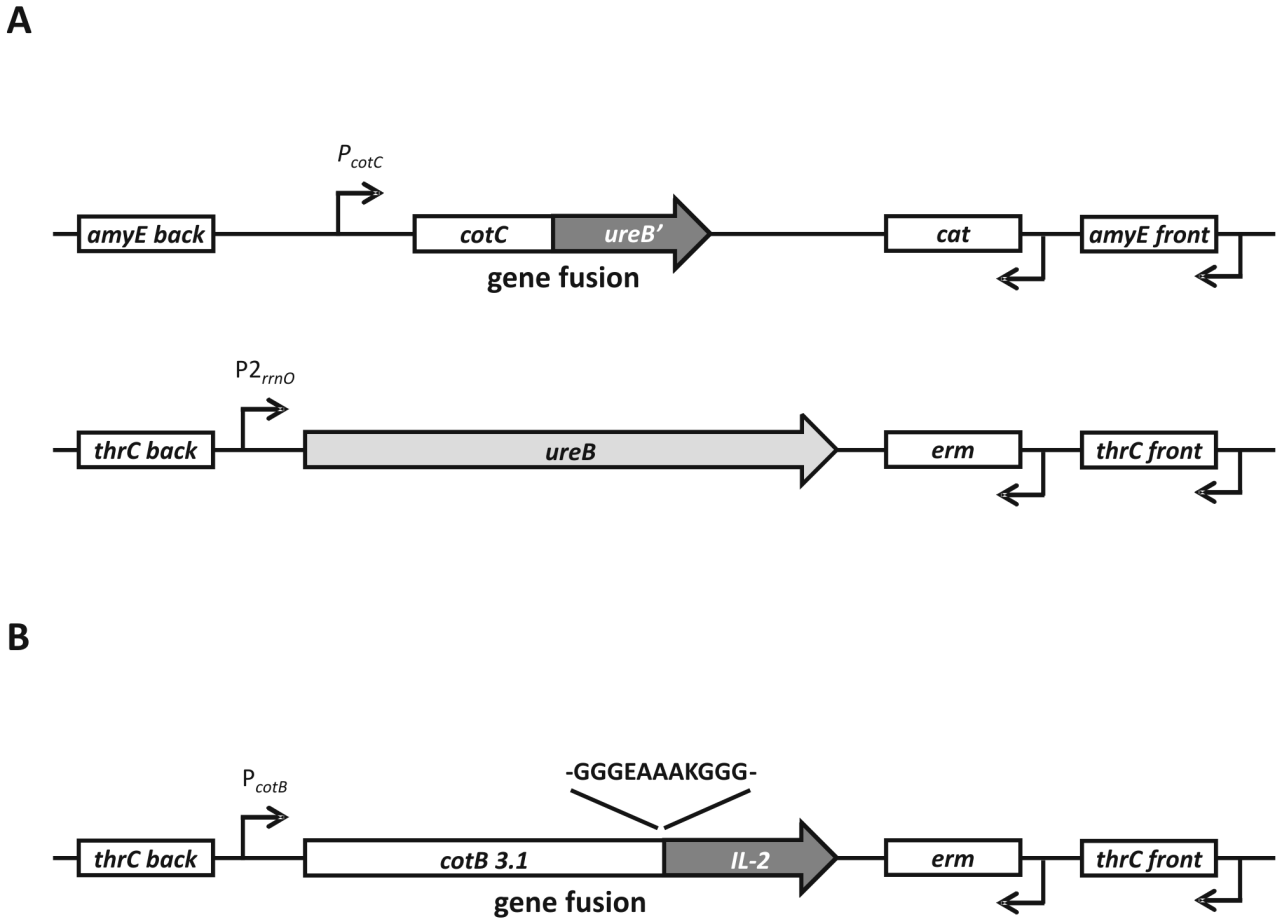
Schematic representation of the three gene fusions constructed. Panel A – gene fusions present in the chromosome of BKH108 strain, Panel B – gene fusion present in the chromosome of BKH121 strain.

In addition, to obtain the expression of full-length UreB in vegetative cells the *ureB* gene was fused to the constitutive promoter of rRNA operon (*rnnOP*) [34] and integrated into *B. subtilis* chromosome at the non-essential locus *thrC* (Fig. 1A).

To achieve recombinant *B. subtilis* spores expressing IL-2 the coding sequence of the *IL-2* gene of *Homo sapiens* was fused in frame with the coding part of *cotB*. The C-terminus of CotB is formed of three 27 amino acid repeats that confer genetic instability to chimeric proteins containing them [31]. For this reason, in case of CotB fusions a fragment of DNA coding for these three repeats was omitted leaving only part of this gene encoding the N-terminal 275 amino acid residues (Fig. 1A). We also added the strong alpha-helix motif (-GGGEAAAKGGG-) [10], [35] between the C-terminus of CotB and N-terminus of IL-2 (Fig. 1B).

The constructed strains were named BKH108 (CotC-UreB, rrnOP2-UreB) and BKH121 (CotB-GGGEAAAKGGG-IL-2) and used for further analysis.

**Figure 2:**
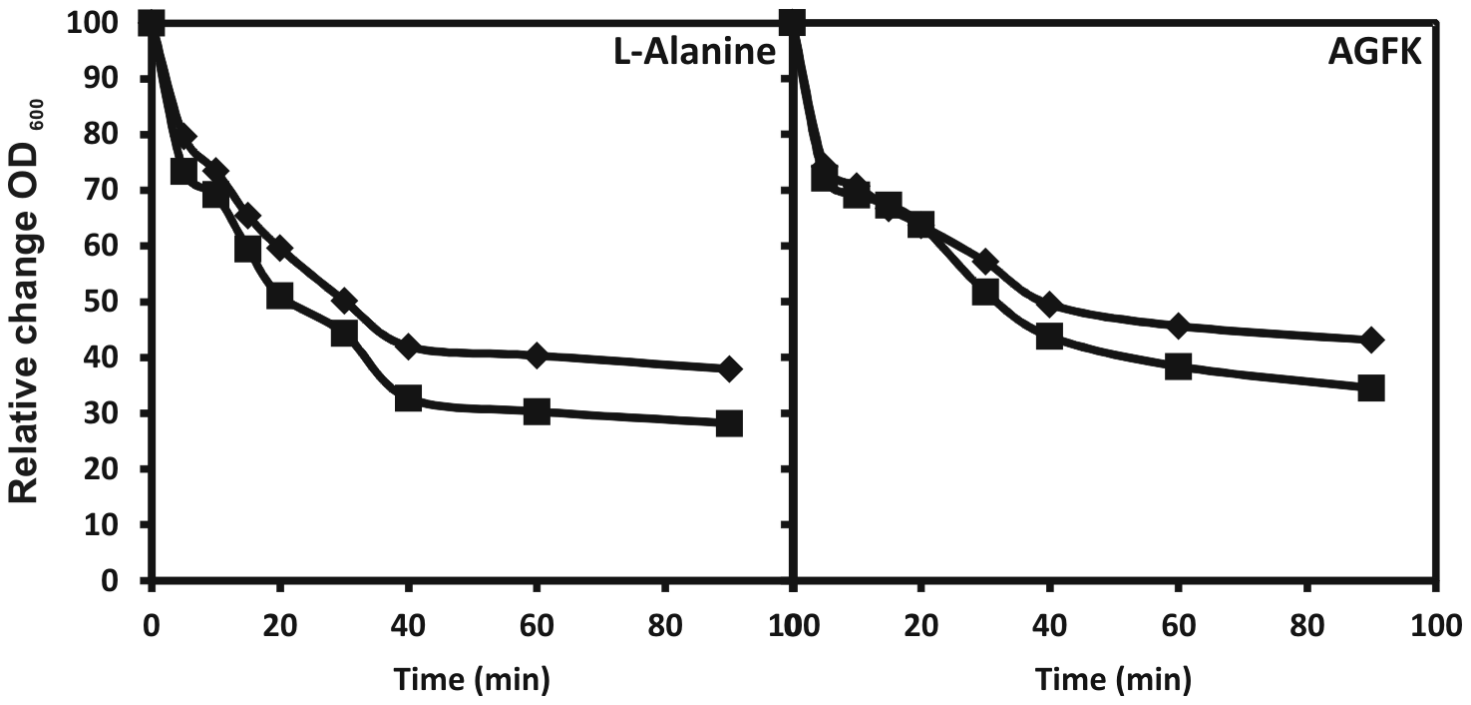
Germination of spore suspensions in l-alanine and AGFK solutions. Spores prepared from cells of 168 (diamonds) and BKH108 (squares) grown in DS medium were heat activated and subsequently incubated in 10 mM Tris-HCl (pH 7.5) with 10 mM l-alanine or with 3.3 mM l-asparaginate, 5.6 mM d-glucose, 5.6 mM d-fructose, and 10 mM KCl (AGFK). Germination was followed by measuring the *A*_600_ of the spore suspension.

The two recombinant strains and their isogenic parental strain 168 showed comparable sporulation and germination (Figure 2) efficiencies and their spores were equally resistant to chloroform and lysozyme treatment (not shown). Therefore, limited to the spore properties that we have analysed, the presence of CotC-UreB and CotB-linker-IL-2 fusions did not affect spore structure or functionality.

### Spore coat expression

**Figure 3:**
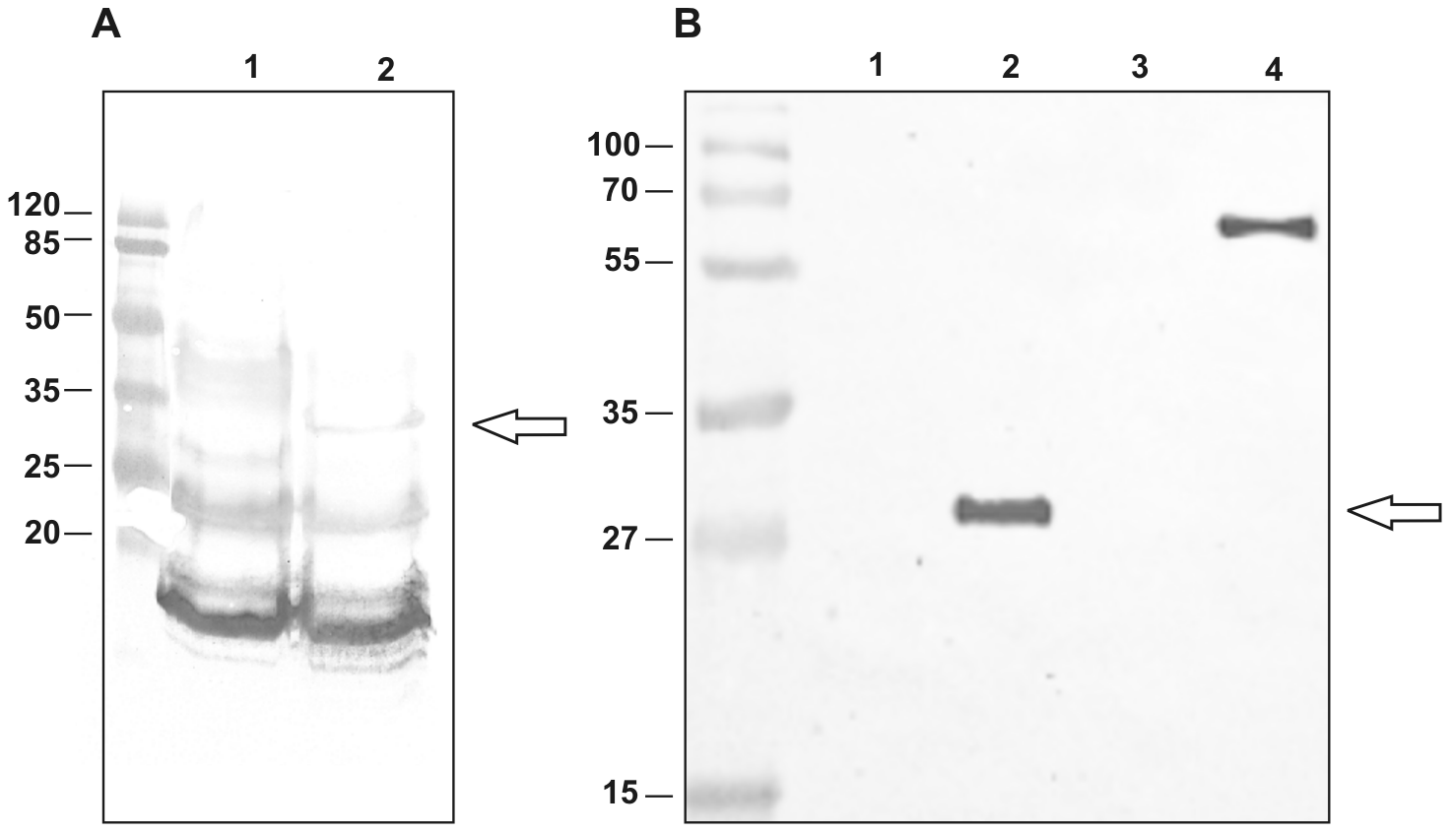
Western blotting analysis of expression of the *cot-ureB* fusion gene and the vegetative expression of the *ureB* gene. Panel A - Spore coat proteins were extracted analysed by western blotting with anti-CotC antibody. Spore coat proteins from spores of the 168 (lane 1) or the BKH108 (fusion CotC-UreB) (lane 2) strain. Panel B – Western blotting with anti-UreB antibody of spore coat proteins from spores of the 168 (lane 1) or BKH108 (fusion CotC-UreB) (lane 2) and of total cell protein extracts of 168 (lane 3) or BKH108 (lane 4) strain. Each lane of panel A and B was loaded with 20 µg of total proteins. Arrows points to fusion proteins.

**Figure 4:**
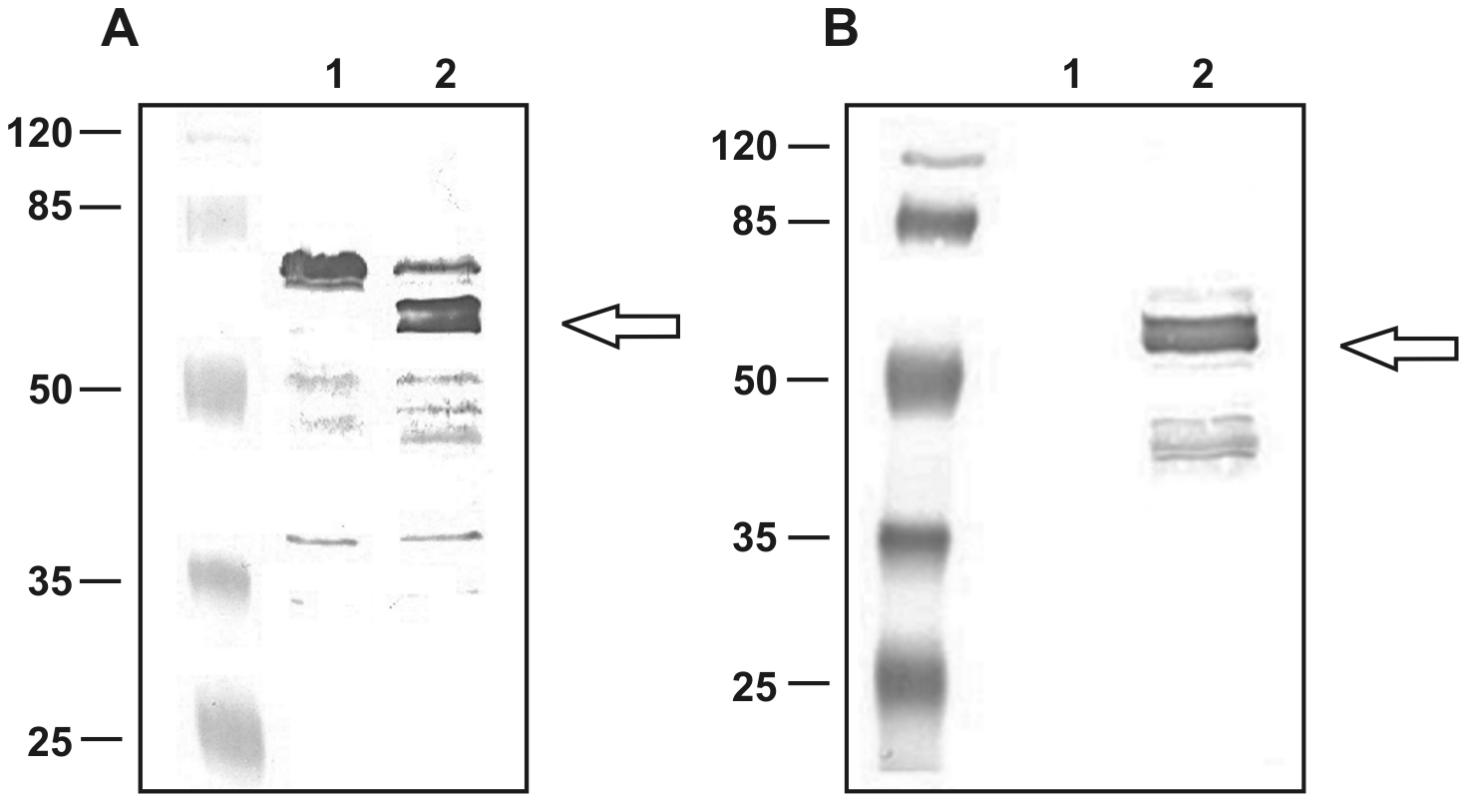
Western blotting analysis of expression of the *cot-linker-IL-2* fusion gene. Western blotting analysis with anti-CotB (Panel A) or anti-IL-2 (Panel B) antibodies. Spore coat proteins from spores of the 168 (lane 1) or BKH122 (fusion CotB-GGGEAAAKGGG-IL-2) (lane 2) strain. Each lane of panel A and B was loaded with 20 µg of total proteins. Arrows point to fusion proteins.

The localization of fusion proteins on the spore coat was tested by western blotting with anti-CotC, anti-UreB, anti-CotB and anti-IL-2 antibodies. The analysis of strain BKH108 showed the presence of an about 28-kDa protein which reacted with both UreB- and CotC-specific antibodies (Fig. 2AB). A standard pattern of CotC and CotU proteins [31], [36] was observed in wild type spores with and without fusion CotC-UreB (Fig. 3A, lanes 1–2). In agreement with a previous report [37], the fusion of a heterologous protein at the C-terminus of CotC impaired the formation of CotC homodimer and CotC-CotU heterodimer. As a consequence, when fused to UreB CotC was only found as a monomer. In addition, the analysis of strain BKH108 showed the presence of an about 62-kDa protein detected by anti-UreB antibodies and corresponding in size to the entire UreB (Fig. 3B, lane 4). As expected this protein was present in extracts from vegetative cells. Western blot analysis of spore coat proteins purified from wild type and recombinant strains BKH121 revealed the presence of an about 55-kDa band which reacted with both IL-2 and CotB-specific antibodies (Fig. 4AB). A 66-kDa band, only reacting with CotB-specific antibody, was present in extracts from wild type and recombinant spores (Fig. 2A), indicating the presence of intact CotB molecules in the spore coat together with CotB-IL-2 fusion protein. A 45-kDa protein, reacting with anti-UreB antibody was observed in strain carrying fusion CotB-linker-IL-2 (Fig. 4B, lane 2). This protein was also recognized by anti-CotB antibody (Fig. 4A, lane 2), we therefore hypothesize that it is a degradation product of CotB-linker-IL-2 fusion.

In both cases the recombinant proteins observed showed apparent molecular weights that correlated well with the deduced molecular weights: Fusion CotC-UreB, 26,9/28; CotB-linker-IL-2, 52.7/55; (deduced/apparent kDa)

### Surface display

**Figure 5:**
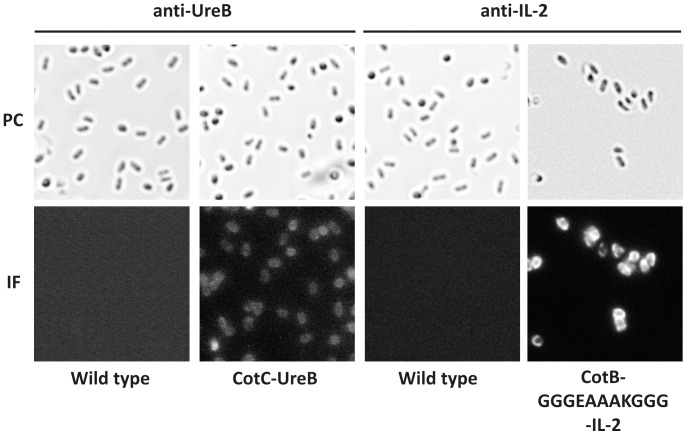
Localisation of fusion proteins as assessed by immunofluorescence microscopy. Purified, free spores of wild type strain 168, BKH108 (CotC-UreB), BKH122 (CotB- GGGEAAAKGGG -IL-2) were visualised by phase contrast (PC) and immunofluorescence (IF) microscopy. The spores were incubated with mouse anti-UreB or anti-IL-2 antibodies, followed by anti-mouse IgG-Cy3 conjugates. The same exposure time was used for all IF images.

The surface localization of CotC-UreB (BKH108) and CotB-GGGEAAAKGGG-IL-2 (BKH121) fusion proteins was analyzed by immunofluorescence microscopy of dormant spores of wild type and recombinant strains using anti-UreB and anti-IL-2 (Abcam, UK) primary antibodies and anti-mouse IgG-Cy3 (Jackson ImmunoResearch Laboratories, Inc). We observed a fluorescent signal around purified dormant spores of both BKH108 and BKH 121 strains (Fig. 5). These results indicate that both fusion proteins are present on the spore coat surface and are available for antibody binding.

### Efficiency of spore surface display

The amounts of CotC-UreB and CotB-GGGEAAAKGGG-IL-2 fusion proteins present on the spore coat surface was assessed using a dot-blotting method. The fusion protein CotC-UreB constituted 0.7% of total spore coat proteins from strain BKH108 (Table 3). From these results, we calculated that the number of fusion protein molecules extracted from a single spore was 1.5×10^4^. In the case of CotB-GGGEAAAKGGG-IL-2 we calculated that the fusion constituted 0.8% of total spore coat proteins, which translates into 9.5×10^3^ molecules of fusion protein per single dormant spore (Table 3).

**Table 3:** Densitometric analysis.

Protein source	Amount of protein used (ng)	Density in OD/mm^2^ (standard deviation)	Protein concentration (ng) in extracts (% of total)	n° of recombinant molecules extracted from each spore
Purified UreB	25.0 ng	172.1 (±0.02)	NA	
	12.5 ng	88,2 (±0.03)	NA	
	6.25 ng	45.7 (±0.12)	NA	
BKH108 (CotC-UreB)	2.50 µg	132.5 (±0.03)	19.25 (0.77)	**1.5×10^4^**
	1.25 µg	67.3 (±0.02)	9.54 (0.76)	
	0.625 µg	34.9 (±0.06)	4.77 (0.76)	
Purified IL-2	25.0 ng	202.6 (±0.01)	NA	
	12.5 ng	102.1 (±0.02)	NA	
	6.25 ng	51.3 (±0.01)	NA	
BKH121 (CotB-linker-IL-2)	2.50 µg	169,6 (±0,01)	20.93 (0.84)	**9.5×10^3^**
	1.25 µg	85,4 (±0,02)	10.45 (0.84)	
	0.625 µg	43,6 (±0,02)	5.31 (0.85)	

### Immune response to recombinant spores

**Figure 6:**
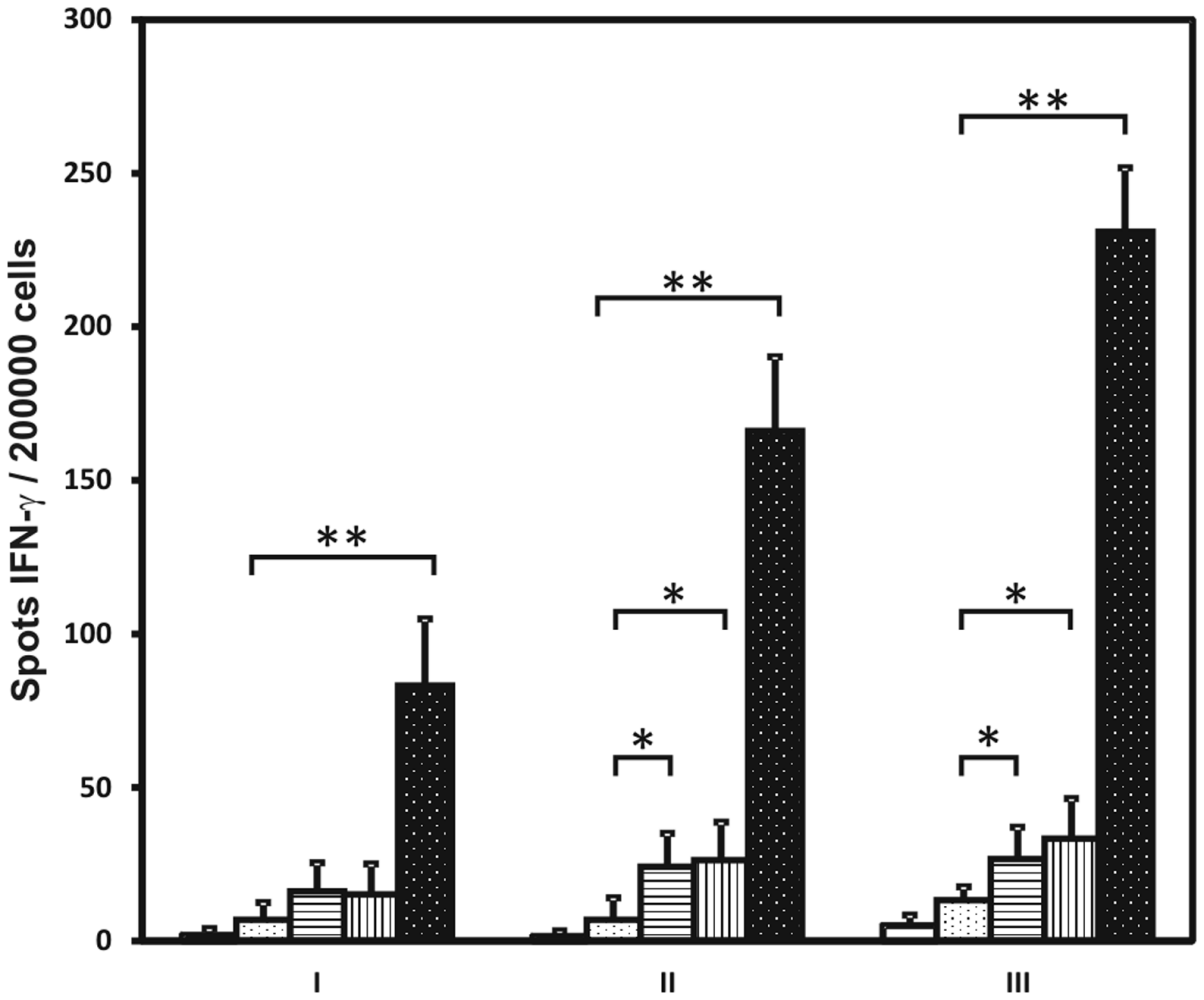
IFN-γ response of sensitized mouse splenocytes to UreB as assessed by the ELISpot. The splenocytes were isolated from naïve mice (open bars), mice orally immunized with 168 spores (dotted open bars), BKH108 (CotC-UreB, vegetative expression of UreB) (bars with horizontal hatching), BKH122 (CotB-linker-IL-2) (bars with vertical hatching) or 1∶1 mixture of BKH108 and BKH122 (dotted closed bars). Cells were treated with purified UreB protein for 72 h and then the IFN-γ cells were enumerated by ELISpot procedure. Error bars represent standard deviation. * p-value <0.05, ** p-value <0.005. I – day 22, II – day 43, III – day 61.

To verify whether such recombinant spores were able to elicit an immune response and whether spores presenting IL-2 acted as adjuvant we orally immunize groups of with spores presenting UreB or IL-2 or with a combination of the two recombinant spores. Production of UreB-specific serum antibody and of IFN-γ and IL-4 by sensitized splenocytes isolated from immunized mice was followed. We were not able to detect UreB-specific antibodies in any of the tested groups of mice (data not shown) suggesting that the immunizations did not induce a humoral response. However, the results of IFN-γ ELISpot experiments revealed that BKH108 spores induced a strong cellular immune response when co-administered with BKH121 spores, presenting human IL-2 (Fig. 6). Moreover, there was clearly visible increase in the immune response along with subsequent immunizations. Interestingly, we were not able to detect IL-4 produced by UreB-sensitized splenocytes isolated from immunized animals (data not shown).

## Discussion

The use of *Bacillus subtilis* spores as mucosal vaccine vehicles has already been tested with various antigens (for review see [4]). The successful applications of spores as vaccines so far reported were based on the use of strong antigens such as tetanus toxin or heat-labile toxin of *Escherichia coli*, that in addition to the strong antigenicity, also serve as efficient adjuvants [38].

Recombinant spores of *B. subtilis* seem to be a perfect choice for *Helicobacter pylori* oral vaccine candidate. Due to unique properties of spores such as heat-stability and ability to safely pass through harsh stomach environment the delivery of *H. pylori* antigens should be much more efficient than in case of other mucosal vaccine systems. Not to be omitted is also the fact that administration of oral vaccines eliminates the need of needles usage and the assistance of trained medical personnel.

We combined three approaches in our design of *H. pylori* spore-based vaccine. First, we used the coat protein CotC as a carrier for a fragment of subunit B of *Helicobacter acinonychis* urease. UreB protein has already been used for immunizations against *H. pylori* infections and is well-characterized antigen of this bacterium [39], [40]. UreB of *H. acinonychis* shares 72% identity with UreB of *H. pylori* and was used to avoid any possible intellectual property issues caused by patents restricting the use of this protein in oral vaccine formulations. BKH108 recombinant spores proved to efficiently display a fragment of UreB protein on the spore surface (Fig. 3A).

*B. subtilis* spores were shown to germinate and most probably undergo re-sporulation inside the gastrointestinal tract (GIT) of laboratory animals [6]. BKH108 spores, apart from presentation of UreB fragment, harbor *ureB* gene of *H. acinonychis* under control of vegetative promoter of *rrnOP* operon. This enables for production of full-length UreB protein in the vegetative cells, which can appear inside the GIT upon germination of spores and increase the amount of antigen in the site of immunization. Indeed, we observed efficient production of UreB protein in BKH108 cells during vegetative growth (Fig. 3B). It is worth notifying, that use of *p_rrnO_* for vegetative expression of an antigen has already been applied for spore-based orally administered vaccines and proved to lead to induction of immune response [41], [42].

An efficient immunization usually requires usage of an appropriate adjuvant. In case of *H. pylori* vaccine such adjuvant should shift the immune response towards Th1 type [43]. Recently IL-2 has been used for that purpose [25]. This cytokine is mainly produced by Th1-polarized helper T-cells and is imposes strong shift towards the cellular immune response [44], [45]. On the other hand, the cellular response has been proposed as a leading one in protecting against *H. pylori* infection [46]. Having in mind these facts we decided to use IL-2-presenting spores, which should serve as an adjuvant helping in the development of immune response. IL-2 has been linked with CotB spore coat protein via previously described linker [47] to additionally improve the display. As a result we obtained spores, which efficiently presented IL-2 (Fig. 4).

The results of oral immunizations of mice suggest that such combination of recombinant spores presenting a fragment of UreB protein and enabling for the expression of this protein in vegetative cells along with IL-2-presenting spores is able to elicit immune response to UreB. The magnitude of response was increasing with each subsequent immunization showing the development of immune memory (Fig. 6). Interestingly, we did not observe production of UreB specific antibodies. However, this observation combined with the lack of IL-4 produced by sensitized mouse splenocytes is not entirely surprising. IL-2, as mentioned above, imposes strong shift towards cellular response. This fact can explain the lack of humoral response to administered antigen. Moreover, when administered alone, BKH108 spores led to the induction of distinct, but statistically significant cellular immune response. In case of IL-2-presenting spores (BKH121) we observed similar phenomenon, which in that case may suggest unspecific induction of immune response by IL-2. This last observation should be verified in further experiments. Nevertheless, when administered together, BKH108 and BKH121 spores led to much stronger induction of cellular immune response suggesting such formulation as a very promising vaccine.

The key question regards a potential risk of IL-2 usage in vaccine formulations. Because of its biological activity an uncontrolled administration of this cytokine may lead to vascular leak syndrome (for review see [48]). Such adverse effect of immunization is undesired therefore it is important to carefully assess safe amount of IL-2 administered along with a vaccine. The formulation of vaccine based on recombinant spores enables for convenient optimization of its composition. In our research we used spores carrying an antigen (UreB) along with spores serving as an adjuvant (IL-2). By changing of proportion of administered spores we can modify the amount of both, an antigen and an adjuvant in final formulation.

In conclusion, *B. subtilis* spores seem to be a promising platform for vaccine candidate against *H. pylori*. Although the application of IL-2 as an adjuvant increases the efficiency of immunization, further research is required to assess protective and therapeutic potentials of such formulation.
